# Treating bipolar disorder in patients with renal failure having haemodialysis: two case reports

**DOI:** 10.1186/1745-0179-4-21

**Published:** 2008-07-26

**Authors:** Maneesh Gupta, Srinivas Annadatha

**Affiliations:** 1 Lancashire Care NHS Foundation Trust, Parkwood Hospital, East Park Drive, Blackpool, FY3 8PW, UK

## Abstract

**Background:**

There is little published guideline or evidence on treating bipolar affective disorder in patients with renal failure having haemodialysis.

**Case:**

We present two patients with bipolar affective disorder with renal failure having haemodialysis. We used lorazepam in one patient to manage the immediate risk of non-engagement with dialysis. Risperidone was added in the second patient for managing psychotic symptoms. Valproate was started as a mood stabiliser and titrated upwards for long-term management of the illness.

**Conclusion:**

We discuss the similarities in the two cases and the care plan we used to manage them.

## Background

Improvements in life expectancy and quality of life in people with chronic medical disorders means new challenges are being created for clinicians. Two decades back chronic renal failure was almost a death knell for patients but today haemodialysis has led to increased life expectancy and increased expectations of patients from their clinicians. For mental health professionals, the increased incidence of psychiatric illnesses in those with chronic illnesses, as well as the increased life expectancy of those with renal failure means that we have to prepare ourselves for treatment of mental health problems in renal failure. While the kidneys are not functioning, and haemodialysis has taken over the renal function, how do we adapt our treatment and manage the concurrent social and health issues.

We present two patients both admitted to an inpatient psychiatric unit, with thrice-weekly haemodialysis and present the treatment challenges and the different needs of these individuals.

### Case 1

A 47-year-old male patient, with end stage renal failure (due to obstructive uropathy) and receiving haemodialysis (for over an year), was admitted. He was known to mental health services and was under a community mental health team. He had been discharged from the inpatient psychiatric unit, two months back, on olanzapine 20 mg at night, which he was partially compliant with.

He had been disruptive and aggressive at the dialysis clinic. He was impatient and often asked for the dialysis to be stopped before the process was over (usually 4 hours). Staff, at his residence, also noticed him to be sleeping less, and not maintaining his diet and fluid restriction. He was often pacing in the common area of the residence and speaking loudly and angrily.

On mental status examination, he was loud, verbally aggressive, demanding and uncooperative. There was flight of ideas, pressure of speech and elevated mood. He understood that he needed dialysis but was overcritical of the time it took for the procedure. The immediate issue to be addressed was facilitating dialysis. Since he consented to having dialysis, the plan was to calm him down so as to allow the entire process to be completed without undue distress to the patient or to others. Olanzapine was reinstated and staff members from the inpatient psychiatric unit accompanied him. He would still be loud, abusive and demanding but would be calmer on verbal de-escalation by staff.

On reviewing his history, a diagnosis of bipolar affective disorder was made and valproate semi sodium started. Valproate has a known role as a mood stabiliser; it is not excreted by the kidney and is affected minimally by haemodialysis in therapeutic concentrations [1]. Dose was gradually (over a period of two weeks) increased to 2 gm per day. Predialysis level was 118 mg/l and 98 mg/l on two occasions. Lorazepam was added at 1 mg twice/day. A further dose of 2 mg before dialysis was prescribed to help the client be calm when in the dialysis room. The use of lorazepam on the morning of dialysis, helped substantially as it was easier to complete the dialysis. After two weeks on valproate 2 gm/day, he was noticed to be calm, friendly, and pleasant. The report from dialysis clinic was that he was sleeping throughout the procedure and was not posing any problems at all. Lorazepam was gradually withdrawn over ten days though a single 2 mg dose was given before dialysis. After a further week without regular lorazepam, the predialysis lorazepam was reduced to 1 mg and then stopped a week later.

No fluctuation was observed in his mental state between the days of haemodialysis and the days when he did not have haemodialysis.

### Case 2

A 44-year-old female patient, known to be suffering from bipolar affective disorder, had been well on lithium for many years. Four years after being on high dose of lithium (1000–1200 mg), consistently high serum lithium levels were noticed. Lithium was tapered down to 200 mg over the next three years. At this stage her urea and creatinine were noticed to be high (urea 10.0 mmol/L, creatinine 345 umol/L). An oral dose of 200 mg lithium daily was resulting in serum lithium levels of 1.16 mmol/L. Hence, lithium was discontinued and valproate semi sodium added at a dose of 500 mg bd. Peritoneal dialysis was started, but was replaced by haemodialysis after one year.

Over these years she had also been receiving lofepramine 140 mg as an antidepressant for emergent symptoms of grief, inability to cope and tiredness. Her renal failure led to a reduction of the dose to 70 mg a day. Valproate was also reduced to 250 mg bd, though the reasons for this are not clear.

She had not needed hospitalization in a psychiatric unit for over 14 years. Recently however, she presented to accident and emergency (A&E) three times within a week with different complaints and physical symptoms, feeling stressed and being fed up of dialysis. She was admitted to the psychiatric unit after her fourth presentation to A&E.

Valproate was increased to 750 mg. On admission, she was difficult, demanding, critical and disruptive. She tried to leave the hospital and refused dialysis. She was detained under the mental health act. During dialysis, she would often shout, 'this is not my machine', 'you are taking all of my blood', or 'I am fed up of this dialysis'. Within the psychiatric unit, she was labile in mood, elevated at times and tearful at others. She continued to express a desire to leave the hospital and not to take medication or have dialysis. Valproate was increased to 1 gm and risperidone was added 1 mg twice a day. Lofepramine was stopped. After four days valproate was increased to 1250 mg daily. She improved within two weeks, became less demanding, calm at dialysis and agreeing to be in hospital.

## Discussion

Renal failure and subsequent haemodialysis restores physiological function for many patients and for many years. Life expectancy for individuals on haemodialysis is increasing especially when they are less than 50 years old [2]. How does it impact on patients with mental health problems? How do psychiatrists titrate the treatment of such patients? This is a new area of psychiatry and the two case reports present similar challenges managed by valproate.

### Diagnosis

Both the patients had a diagnosis of mental illness before chronic renal failure set in and before haemodialysis was started. The first patient had been on the psychiatric ward a few weeks before this presentation and discharged, as he was well. The second patient was being managed by the community mental health team (CMHT) for over fourteen years, with a mood stabiliser and an antidepressant. During this episode, a possibility of a mixed episode was considered.

### Symptoms

Both patients were distressed with dialysis though they understood that it was a life saving procedure. It is noteworthy that despite the nature and degree of mental health problems, both had not missed any appointment for dialysis. The time it takes during the process was difficult for both patients; and during their exacerbations a common theme was of demanding to be released off dialysis sooner than the complete four/three hours respectively. This is in keeping with the increased psychomotor activity seen in mania.

### Blood investigations

Patients on dialysis have monthly blood tests during the dialysis procedures and this eliminated the need for the ward to arrange for blood tests. It was arranged for copies of the reports to be sent to us.

### Pharmacological treatment

Treatment was chosen to be valproate as it is metabolised in the liver and only 1% is excreted through the kidney [1]. Dosage adjustments are not required in renal failure [3]. It is thus a safe choice to prescribe for an individual with renal failure.

Haemodialysis clears toxins that are < 500 g/mol, are water soluble, have small volumes of distribution, follow single compartment kinetics and are poorly bound to plasma proteins. Valproate is a small (144 Da), water-soluble molecule with a volume of distribution of only 0.1–0.4 l/kg. However, at therapeutic doses it is 90–95% bound to plasma proteins [1,4]. At toxic doses, the plasma protein binding decreases (as a percentage) and it no longer demonstrates single compartment kinetics. Hence, haemodialysis is now recommended in valproate overdose [1]. Although haemodialysis has been shown to reduce the level of valproate, but this has not been a substantial difference at therapeutic levels [1]. There is however, no guideline on using valproate as a therapeutic tool while a patient is on haemodialysis.

The other two mood stabilisers lithium and carbamazepine were not considered because of renal failure being a relative contraindication. A review of treatment of patients with bipolar disorder and medical comorbidity [5], suggested use of lithium in a patient on haemodialysis [6] in a single post-dialysis dose of 300–600 mg and discussed a case report of intraperitoneal lithium during CAPD [7]. Carbamazepine is to be avoided due to reduced clearance of its toxic metabolite [5].

The first case also highlights the relative lack of efficacy of olanzapine alone in maintaining remission. The use of lorazepam in him is a treatment modality we successfully used. It worked well helping the dialysis process to proceed as scheduled while the mood stabiliser was titrated and had a therapeutic effect. Lorazepam is not recommended in renal failure, but haemodialysis has been found to reduce the peak plasma concentration, T*max *and half life [8], thus posing no increased risk.

In the second patient we chose to add risperidone, due to congruent psychotic symptoms. Risperidone is not recommended in renal failure [5] and there is no data on its use in a patient with haemodialysis. We therefore kept the daily dose as low as was necessary to achieve clinical response.

### Nursing need

During hospital stay, a staff member was identified who would accompany the patient to the dialysis unit. This was to ensure that the dialysis process was not impeded or missed. This staff member was trained in verbal descalation of the patient while in the dialysis unit and on the machine itself.

Patients on renal failure and having haemodialysis are managed by diet restrictions and medication that reduces the toxins that accumulate in the body. These additional components of our patients' management meant developing a specific nursing care plan and carefully overseeing the implementation of this plan. Changes to medication and responding to concerns of the renal physician and other health professionals was also required. A printed information booklet was kept in the ward office for all staff to familiarise themselves with renal failure, dialysis and renal transplant.

### Discharge planning

The first patient was living in 24 hr supported accommodation, but after two admissions, they were reluctant to have him back. While we attempted to resolve the concerns of the staff and management of the supported housing, we also had to search for other options. A trained worker was provided for accompanying him to dialysis three times a week, from his supported accommodation. The second patient had strong family support and involvement. They were very happy to have her back.

### Transplant list

The first patient has not been included on a transplant list, because of concerns about his capability to monitor and manage medication, and the prognosis of his mental health problem. The second patient was on a transplant list but was taken off it, due to her hospitalization in a psychiatric unit. We are still trying to resolve this issue.

## Conclusion

Patients with bipolar disorder and renal failure having haemodialysis require treatment in a psychiatric inpatient unit to be tailored to their unique needs. We have presented two cases that were managed with almost similar care plans and medication. It is hoped that other mental health professionals faced with similar patients will find our approach informative and will contribute to the published literature on this challenging clinical scenario.

## Competing interests

Dr Maneesh Gupta has accepted hospitality from various pharmaceutical companies in UK. He has accepted honoraria to speak at clinical gatherings from Astra Zeneca and Wyeth Laboratories. He holds some shares in some Indian pharmaceutical companies.

Dr Srinivas Annadatha has accepted hospitality of pharmaceutical companies in the UK.

## Authors' contributions

MG conceived of the paper. MG drafted the manuscript, reviewed the literature and provided care to the first patient. SA contributed to the data collection, review of literature and provided care to the second patient.

Both authors read and approved the final manuscript.
